# Integrated Care Components in Transitional Care Models from Hospital to Home for Frail Older Adults: A Systematic Review

**DOI:** 10.5334/ijic.6447

**Published:** 2022-06-29

**Authors:** Merel Leithaus, Audrey Beaulen, Erica de Vries, Geert Goderis, Johan Flamaing, Hilde Verbeek, Mieke Deschodt

**Affiliations:** 1 Academic Center for Nursing and Midwifery, Department of Public Health & Primary Care, KU Leuven, BE; 2 Department of Health Services Research, Maastricht University, NL; 3 Living Lab in Ageing and Long-Term Care, Maastricht, the Netherlands; 4 Academic Center for General Practice, Department of Public Health & Primary Care, KU Leuven, BE; 5 Gerontology and Geriatrics, Department of Public Health & Primary Care, KU Leuven, BE; 6 Division of Gerontology and Geriatrics, University Hospitals Leuven, BE; 7 Competence Center of Nursing, University Hospitals Leuven, BE

**Keywords:** integrated care, frail older adult, transitional care, systematic review

## Abstract

**Introduction:**

Frail older adults frequently experience transitions from hospital to home due to their complex care needs. Transitional care models (TCMs) are recommended to tackle adverse outcomes in frail patients. This review summarizes the use of integrated care components in addressing transitional care from hospital to home, provides an overview on reported outcomes and describes the impact of identified components on the outcomes hospital readmission and emergency department visit.

**Methods:**

This study is part of the European TRANS-SENIOR project. PubMed, CINAHL and Embase were searched for studies in English, German and Dutch that describe a TCM for frail older patients including both pre- and post-discharge components.

**Results:**

Seventeen studies, covering 15 TCMs were included. All TCMs describe a person-centred, tailored, pro-active and continuous transitional care service. Components like a small sized care team, intensive follow-up, shared decision making and informal caregiver involvement are likely to be associated with reduced hospital readmission and ED visits. Twenty-seven transitional care outcomes were reported: 19 service outcomes, six patient outcomes and two provider outcomes.

**Conclusion:**

Heterogeneity in content and outcomes complicates between-study comparison, yet several components were identified that improved care outcomes. Patient and provider outcomes should be included in future research.

## Introduction

Frail older adults represent between 25 and 50 % of the population 85 years or older [1]. They have a high risk to be admitted to the hospital due to their complex profile characterized by multimorbidity, polypharmacy and biopsychosocial needs [2]. In addition, multimorbidity, impaired functionality, older age, poor social support and history of depression increases the risk of hospital readmissions [3]. Hence, frail older adults frequently experience care transitions between the hospital and their home. Besides the risk factors related to hospital readmission, the transitional care process itself can cause further harm. Research has shown that poor transitions can lead to adverse outcomes like poor clinical outcomes, missed diagnosis or incorrect treatment, dissatisfaction among patients, inappropriate use of healthcare services, rehospitalization and mortality [4]. Results of a prospective cohort study showed that within five weeks of discharge 19% of patients experienced an adverse event of which one third was considered preventable [5].

Transitional care interventions have been recommended to tackle adverse outcomes in frail older people with complex care needs. Transitional care is defined as “a set of actions designed to ensure the coordination and continuity of healthcare as individuals transfer between different locations or different levels of care within the same location” [6, p.4]. Transitional care models (TCMs) within integrated settings can particularly benefit frail older patients as they often have various health care needs and undergo frequent transitions. Integrated care is defined as “the management and delivery of health services so that clients receive a continuum of preventive and curative services, according to their needs over time and across different levels of the health system” [7, p.1]. Integrated care aims to improve patient, care provider and healthcare service outcomes by increasing the quality of care and decreasing health care costs. Transitional care is part of integrated care as it often occurs during longer care episodes and within long-term chronic disease management [8].

A meta-review including 17 reviews published between 1950 and 2014 identified that included studies provided limited descriptions of the health system context and concluded that transitional care interventions in integrated health system settings have not been well studied [9]. The reviewers indicated that successful transition interventions tend to address common aspects of the care transition including intervention components before and after hospital discharge, and tailored care based on individual patient needs. In a recent review that describes components of TCMs in geriatric patients, Morkisch et al. [10] added that staffing (multidisciplinary team), assessing/managing symptoms, educating/promoting self-management, maintaining relationships and fostering collaboration seem to be influential in reducing readmission rates.

Also Linertová et al. [11] concluded that transitional care interventions including some type of home care during follow-up were more successful in reducing readmissions than those without follow-up [11]. Similarly Allen et al. [12] suggested that successful transitions should include both hospital discharge planning and in-home follow-up. The latter review also highlighted the wide variety of outcomes measured in TCM research. While hospital readmission and length of stay was measured in all studies, patient satisfaction was assessed in only half of the studies, and other patient and family centred outcomes where scarce. Caregiver satisfaction was not assessed in any study [12].

Although several reviews have described the impact of transitional care for older patients on hospital readmission rates, this is the first review that will focus on the use of integrated care components in addressing transitional care for frail older patients from hospital to home. To describe the use of integrated care components we will use the SELFIE framework (Sustainable intEgrated chronic care modeLs for multi-morbidity: delivery, FInancing, and performancE) by Leijten et al. (2018). The framework structures relevant concepts of integrated care for persons with multi-morbidity. Implementation of TCMs that include integrated care components can address the complex care needs and improve the quality of the transition from hospital to home of frail older patients. This systematic review therefore aims to 1. identify integrated components used within TCMs from hospital to home for frail older patients and map these components to the SELFIE framework, 2. list the service, patient and provider outcomes measured in the included studies and 3. map TCM components that effectively reduce the risk of hospital readmissions and emergency department (ED) visits for older patients.

## Methods

The systematic literature review was reported following the PRISMA guideline. The study protocol was registered in the PROSPRO database (CRD42020208434).

### Search strategy

A systematic database search was conducted in PubMed, Embase and CINAHL to identify eligible studies. The search strategy was limited to articles in English, German and Dutch and published between January 2000 and June 2020. Main concepts were defined and synonyms were searched to create a comprehensive search string. All search terms used for the electronic databases are available in the additional file 1. Further studies were included through a reference list search of the included studies.

### Eligibility criteria

Intervention studies were eligible if they included participants of 65 years or older with a frailty profile. This could be a decline in one or more functional domain(s) or the presence of one or more chronic disease(s). Studies were included if they described a TCM focusing on improving transitional care between the hospital and the home. The TCMs had to include both a pre-discharge intervention component in the hospital and a post-discharge follow-up component to show a clear link between the hospital and follow-up setting. Finally, studies had to report at least on hospital readmission or ED visit rates as an outcome.

Studies with a descriptive or qualitative study design were excluded, as well as studies describing an intervention conducted only at the ED or when patients were not hospitalized. In addition, palliative care interventions and interventions that focused on other care transitions than hospital to home were excluded.

### Slection process

After removing duplicates, all titles and abstracts were screened independently by two out of three available reviewers (ML, AB, EV). In case one of the reviewers scored the publication as potentially relevant, it proceeded to the next review stage, where a full-text screening was performed by one researcher (ML). A second researcher (AB) supported the full-text screening of uncertain articles to assess eligibility. In case of disagreement, publications were discussed with a third reviewer (MD) to reach consensus. The study selection was performed using the Rayyan application for systematic reviews [13].

### Data extraction and synthesis

Data was extracted by one reviewer (ML) and checked by a second reviewer (MD). A data extraction form was created and tested with two of the included articles. Adjustments were made as needed. Extracted data included general study characteristics (year of publication, study design, objectives), participant characteristics (age, gender, frailty profile), intervention characteristics (setting, duration, description of the pre-discharge and post-discharge intervention components, involved care providers), characteristics of intervention and control group (total participants, total participants included in analysis, loss of follow-up, length of follow up), control overview (setting and description of usual care or control care), and outcomes (hospital readmission and ED visit rate, secondary outcomes, process outcomes).

Data synthesis was conducted using the SELFIE framework [14]. The framework describes key elements of integrated care for multi-morbidity by grouping components around the six adapted WHO core health systems elements: service delivery, leadership & governance, workforce, financing, technologies & medical products, and information & research. The components are sorted within the micro (e.g. patient and multidisciplinary teams), meso (e.g. organizational & structural development) and macro level (e.g. policy and political commitment). In the data synthesis the SELFIE framework was used to map the TCM components according to the micro level as information on meso and macro level was rarely reported (Table 2). We applied the descriptions and examples provided by Leijten et al. (2018) to map the integrated care components used in the TCMs. The mapping of components was conducted by one reviewer (ML) and in case of doubt discussed with a second reviewer (MD) until consensus was reached.

Extracted data was summarized in four tables. Table 1. captures the study characteristics of the included studies. Table 2. summarizes the TCM components mapped to the micro level of the SELFIE framework. Table 3. categorizes all outcome measures by service, patient and provider outcomes and ranks them to frequency of reporting. Table 4. provides an overview of the impact of the TCMs on hospital readmission and ED visits rates. As reporting the impact of TCMs on hospital readmission and ED visits was a secondary objective, this review did not perform a risk of bias assessment.

**Table 1:** Study characteristics of included studies.


STUDY	COUNTRY	STUDY DESIGN	TCM NAME	SAMPLE SIZE	MEAN AGE (YEARS)	INCLUSION CRITERIA

Brand (2004)	Australia	Quasi-experimental	/	IG: 83 CG: 83	IG: 77.5 CG: 79.6	≥65 years Inpatient stay >24 hours At least one risk criteria: admission in past six months, two or more actively treated comorbidities, admitted because of chronic heart failure

Buurman (2016)	Netherlands	RCT	Transitional Care Bridge Intervention	IG:316 CG:303	IG:79.7 CG:80.0	≥65 years At least 48 hours admitted to internal medicine At risk for functional decline risk score based on the Identification of Seniors at Risk Hospitalized Patients score

Coleman (2004)	US	Quasi-experimental	The Care Transitions Intervention	IG:158 CG:1235	IG:75.1 CG:78.4	≥65 years Community-dwelling At least one of nine diagnoses: congestive heart failure, chronic obstructive pulmonary disease, coronary artery disease, diabetes mellitus, stroke, medical and surgical back conditions (predominantly spinal stenosis), hip fracture, peripheral vascular disease, and cardiac arrythmias

Coleman (2006)	US	RCT	The Care Transitions Intervention	IG:376 CG:371	IG:76.0 CG:76.4	≥65 years Community-dwelling Admitted for nonpsychiatric condition No documentation of dementia No plans to enter hospice At least one of 11 diagnoses: stroke, congestive heart failure, coronary artery disease, cardiac arrhythmias, chronic obstructive pulmonary disease, diabetes mellitus, spinal stenosis, hip fracture, peripheral vascular disease, deep venous thrombosis, and pulmonary embolism

Courtney (2009)	Australia	RCT	Older Hospitalised Patients’ Discharge Planning and In-home Follow-up Protocol (OHP-DP)	IG:49 CG:58	IG:78.1 CG:79.4	≥65 years Admitted with a medical diagnosis At least one risk factor for readmission: ≥75 years, multiple admissions in previous 6 months, multimorbidity, living alone, lack of social support, moderate to severe functional impairment, history of depression

Gregersen (2012)	Denmark	Retrospective design with two historical cohorts	/	IG:233 CG:262	IG:82.6 CG:82.1	≥65 years Admitted to the orthopaedic department Primary diagnosis of hip fracture

Huckfeldt (2019)	US	Quasi-experimental	Safe Transitions for At Risk Patients (STAR)	IG:202 CG1: 4142, CG2: 4592	/	≥75 years With one or more defined conditions: hospital admission in past 30 days, altered mental status or dementia, fall or syncope, volume depletion, dehydration, acute kidney injury, shortness of breath, generalized weakness, failure to thrive

Lembeck (2019)	Denmark	RCT	/	IG:270 CG:267	IG:82.5 CG:82.2	≥65 years Minimum three out of nine defined medical and social conditions: cognitive and psychiatric disorders, drug and alcohol abuse, lack of social network, low level of functioning, multiple medications, hospital contacts in previous six months, falls history, housing conditions that hamper the patient in his daily activities.

Lim (2013)	Australia	RCT	Post- Acute Care (PAC) Intervention	IG:311 CG:287	IG:76.5 CG:76.8	Admitted to acute ward for over 48 hours, Discharged home Expected to live at least one month post discharge Requiring community services at discharge Mobility or self-care management problem OR fulfilling two or more defined factors: living alone, taking care of other at home, using community services before admission

Naylor (2004)	US	RCT	Discharge planning and home follow-up protocol	IG:118 CG:121	IG:76.4 CG:75.6	≥65 years Community-dwelling Heart failure diagnosis Being alert and oriented

Ornstein (2011)	US	Pre-post design	/	IG:532 CG:628	IG:81.1 CG:/	Not reported

Parry (2009)	US	RCT	The Care Transitions Intervention	IG:44 CG:42	IG:80.5 CG:82.8	≥65 years Community-dwelling Admitted for nonpsychiatric condition No documentation of dementia No plans to enter hospice At least one of 11 diagnoses: stroke, congestive heart failure, coronary artery disease, cardiac arrhythmias, chronic obstructive pulmonary disease, diabetes mellitus, spinal stenosis, hip fracture, peripheral vascular disease, deep venous thrombosis, and pulmonary embolism

Rebello (2017)	US	Retrospective analysis of a clinical demonstration	The PILL program	IG:100 CG:100	IG:74.5 CG:74.4	≥65 years Acute admission and discharged home Lived in rural or highly rural area Patients fulfilling one or more defined risk factors were prioritized: ≥75 years, polypharmacy, cognitive impairment, congestive heart failure

Shakib (2016)	Australia	Retrospective case-control study	Multidisciplinary Ambulatory Consulting Service (MACS)	IG:252 CG:1008	IG:77.0 CG:77.0 (median years)	≥65 years Two or more chronic conditions At least two MACS clinic visits

Simpson (2019)	US	Matched case-control study	Bundled Help (Hospital Elder Life Program)	IG:148 CG:148	/	≥65 years At least one risk factor for ADL impairment, vision and/or hearing impairment, or dehydration

Villars (2013)	France	Quasi-experimental before and after design	/	IG1: 222, IG2: 168 CG:/	IG:81.8 CG:/	Hospitalized in the Special Alzheimer Acute Care Unit At least one of the emergency room re-hospitalization risks: severe disruptive BPSD, change of living arrangement related to BPSD, exhaustion of the principal caregiver, discharge with anosognosia while living alone in the community

Wee (2014)	Singapore	Retrospective cohort study	Aged Care Transition	IG:4132 CG:4132	IG:79.2 CG:79.1	At least one of the following criteria: ≥65 years, multimorbidity, ≥ five medications, impaired mobility or functional decline, impaired self-care skills, poor cognitive status, catastrophic injury, chronic illness, living alone or poor social support, multiple hospitalizations or ED visits in last six months


**US** = United States, **RCT** = Randomised controlled trial.

**Table 2:** TCMs mapped to the micro level of the SELFIE framework.


	SERVICE DELIVERY							LEADERSHIP & GOVERNANCE			WORKFORCE			FINANCING			TECHNOLOGIES & MEDICAL PRODUCTS			INFORMATION & RESEARCH		
					
	PERSON-CENTRED	TAILORED	SELF-MANAGEMENT	PRO-ACTIVE	INFORMAL CAREGIVER INVOLVEMENT	TREATMENT INTERACTION	CONTINUITY	SHARED DECISION-MAKING	INDIVIDUALIZED CARE PLANNING	COORDINATION TAILORED TO COMPLEXITY	MULTI-DISCIPLINARY TEAM	NAMED COORDINATOR	CORE GROUP	COVERAGE & REIMBURSEMENT	OUT OF POCKET COSTS	FINANCIAL INCENTIVES	EMRS & PATIENT PORTALS	E-HEALTH TOOLS	ASSISTIVE TECHNOLOGIES	REMOTE MONITORING	INDIVIDUAL LEVEL DATA	INDIVIDUAL RISK PREDICTION

	Studies with a significant impact on hospital readmissions and/or ED visits																					

Coleman (2004 & 2006), Parry (2009)	x	x	x	x	x	x	x	x	x	x		GNP	/	x							x	x

Courtney (2009)	x	x	x	x	x	x	x	x	x	x		RN	RN, physio-therapist						x		x	x

Naylor (2004)	x	x	x	x	x	x	x	x	x	x	x	APN	APN, physicians						x		x	x

Rebello (2017)	x	x	x	x	x	x	x		x	x		PILL pharmacist	PILL pharmacist, Pill program manager				x				x	x

Wee (2014)	x	x	x	x	x	x	x	x	x	x		RN or MSW	RN/MSW, project director, clinician leader								x	x

	Studies with a non-significant impact on hospital readmissions and/or ED visits																					

Brand (2004)	x	x	x	x		x	x		x	x	x	CDNC	CDNC, GP								x	x

Buurman (2016)	x	x	x	x	x	x	x	x	x	x	x	CCRN	CCRN, RN, geriatrician								x	x

Gregersen (2012)	x	x	x	x		x	x		x	x	x	/	geriatrician, physio- therapist, nurse				x				x	x

Huckfeldt (2019)	x	x	x	x	x	x	x		x	x	x	HHN	HHN, geriatrician, nurse								x	x

Lembeck (2019)	x	x		x		x	x		x	x		PN	PN, MN, DN	x							x	x

Lim (2013)	x	x		x			x		x	x		Allied health staff or nurse	/	x							x	x

Ornstein (2011)	x	x	x	x	x	x	x		x	x	x	NP	NP, PCP, inpatient care team	x			x		x		x	x

Shakib (2016)	x	x	x	x		x	x	x	x	x	x	/	/				x		x		x	x

Simpson (2019)	x	x	x	x		x	x		x		x	Geriatrician or NS or ELS	ELNS, RN, CNA, PT, OT, ST				x					x

Villars (2013)	x	x		x	x	x	x	x	x	x	x	/	/								x	x


**CDNC** = chronic disease nurse consultant, **CCRN** = community care registered nurse, **RN** = registered nurse**, APN** = advanced practice nurse, **NP** = nurse practitioner, **NS** = nurse specialist, **GNP** = geriatric nurse practitioner, **HHN** = Home health nurse, **MN** = municipal nurse, **PN** = project nurse, **DN** = Discharging nurse, **ELNS** = elder life nursing specialist, **ELS** = elder life specialist, **CNA** = certified nursing assistant, **PT** = physical therapist, **OT** = occupational therapist, **ST** = speech therapist, **MSW** = medical social worker, **EMR** = electronic medical record.

**Table 3:** Outcome measures categorized by service, patient, provider and process outcomes and ranked based on frequency of reporting.


CATEGORY	OUTCOME VARIABLE	# OF STUDIES REPORTED	% OF REPORTED STUDIES	FIRST REPORTED STUDY YEAR	LAST REPORTED STUDY YEAR	CONTINENT		

						US	EU	APAC

Service outcomes	Hospital readmission	17	100%	2004	2019	8	4	5

	ED visits	9	53%	2004	2019	4	1	4

	Average length of hospital stay	6	35%	2004	2019	3	1	2

	Total intervention cost	6	35%	2004	2017	5	0	1

	Health care cost	4	24%	2004	2013	3	0	1

	GP visits	3	18%	2004	2019	0	1	2

	Other community service use	3	18%	2013	2019	1	1	1

	Discharge destination	3	18%	2004	2016	1	1	1

	Total number of hospital days	2	12%	2004	2019	1	1	0

	Time to first unplanned re-hospitalization	2	12%	2004	2016	2	0	0

	Rehospitalization for same diagnosis as index hospitalization	2	12%	2006	2009	2	0	0

	Proportion of preventable readmissions	1	6%	N/A	2019	0	1	0

	Minutes per day among patients receiving municipal services	1	6%	N/A	2019	0	1	0

	Change in minutes per day before to after discharge	1	6%	N/A	2019	0	1	0

	New fracture	1	6%	N/A	2009	0	1	0

	Time to first ED visit	1	6%	N/A	2004	1	0	0

	Complicated posthospital episode	1	6%	N/A	2004	1	0	0

	Average Number of readmissions following discharge from index admission	1	6%	N/A	2016	1	0	0

	Case-mix index	1	6%	N/A	2011	1	0	0

Patient outcomes	Mortality	10	59%	2004	2019	3	4	3

	Health related Quality of life	5	29%	2004	2013	1	1	3

	Activities of daily living	2	12%	2013	2016	0	2	0

	Functional status	2	12%	2004	2016	1	1	0

	Patient satisfaction	2	12%	2004	2014	1	0	1

	Personalized health goal	1	6%	N/A	2009	1	0	0

Provider outcomes	Caregiver burden	1	6%	N/A	2013	0	0	1

	Provider feedback	1	6%	N/A	2011	1	0	0

Process outcomes	Reported process outcome	13	76%	2004	2019	7	3	3


**APAC** = Asia-Pacific (including Australia), **EU** = Europe, **US** = United States, **N/A** = not applicable, **GP** = general practitioner, **ED** = emergency department.

**Table 4:** Impact of TCMs on hospital readmission and ED visit outcomes.


	HOSPITAL READMISSION (%)					ED VISITS (%)			
	
	LESS THAN 1 MONTH	1 MONTH	2–3 MONTHS	5–6 MONTHS	1 YEAR	LESS THAN 1 MONTH	1 MONTH	2–3 MONTHS	5–6 MONTHS

Brand (2004)			IG: 36.1 CG: 36.1	IG: 31.3 CG: 25.3				IG: 8.4 CG: 8.4	IG: 21.7 CG: 18.1

Buurman (2016)				IG: 33.5 CG:29.0					

Coleman (2004)		IG: 8.9 CG: 13.8	IG: 13.5 ** CG: 22.0	IG: 22.9 * CG: 32.0			IG: 11.0 CG: 14.2	IG: 18.3* CG: 25.7	IG: 37.1 CG: 36.0

Coleman (2006)		IG: 8.3* CG: 11.9	IG: 16.7* CG: 22.5	IG: 25.6 CG: 31.0					

Courtney (2009)				IG: 22.0** CG: 46.7					IG: 28.5 CG: 46.5

Gregersen (2012)			IG: 13 CG: 12	IG: 27 CG: 26					

Huckfeldt (2019)	IG: 5.9 CG1: 4.9 CG2: 5.9	IG: 18.3 CG1: 14.3 CG2: 14.6				IG: 5.0 CG1: 2.9 CG2: 3.2	IG: 10.9 CG1: 7.2 CG2: 7.9*		

Lembeck (2019)	IG: 11 CG: 10	IG: 30 CG: 26		IG: 56 CG: 54					

Lim (2013)				IG: 25 CG: 28					IG: 6 CG: 6

Naylor (2004)					IG: 104 (n)* CG: 162 (n)				

Ornstein (2011)			IG: 15.7 CG: 16.6						

Parry (2009)		IG: 6.8 CG: 16.7	IG: 9.3** CG: 31.0	IG: 20.9 CG: 38.1					

Rebello (2017)		IG: 10 CG: 10	IG: 13 CG: 15				IG: 7 CG: 20 OR = 0.30 (.12–.75)	IG: 16 CG: 26	

Shakib (2016)					IG: 21 CG: 23.9				

Simpson (2019)		IG: 16.8 CG: 28.4					IG: 10.8 CG: 15.5	IG: 10.8 CG: 15.5	

Villars (2013)		IG1: 13.31 IG2: 13.19 CG: 16.07	IG1: 24.03 IG2: 23.58 CG: 28.98						

Wee (2014)	IG: 10.0*** CG: 21.3	IG: 15.6*** CG: 27.7	IG: 15.6*** CG: 27.7	IG: 37.9** CG: 51.6			IG: 19.3*** CG: 32.0		IG: 46.3* CG: 57.9


***= p ≤ .001, **= p ≤ .01, *= p ≤ .05, **OR** = Odds Ratio, **(n)** = number.

## Results

### Study selection

**Figure 1:**
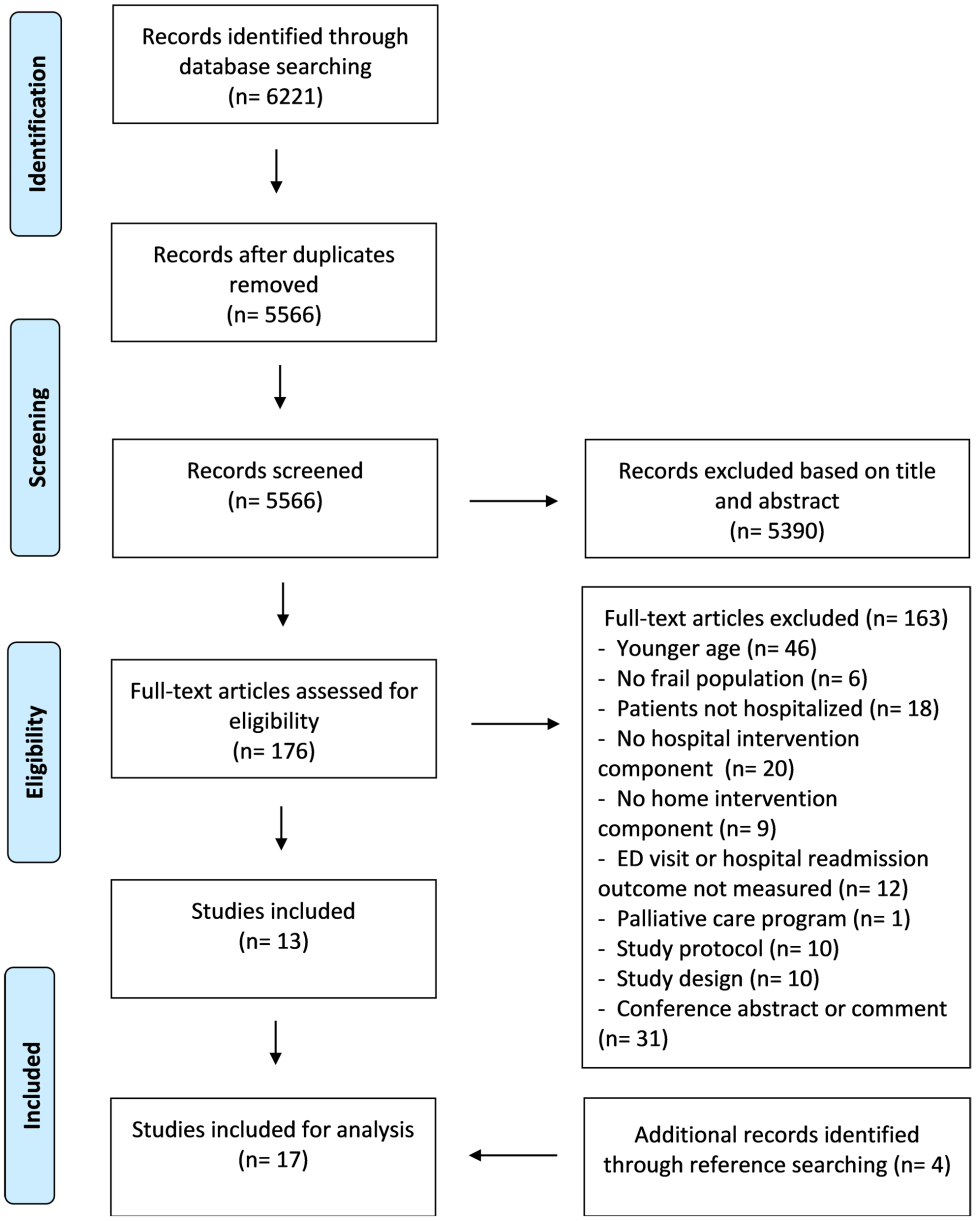
Overview of the screening and selection process using the PRISMA flow chart.

The search strategy identified 6221 records. After removing duplicates, title and abstract of 5566 records were screened. Of the 176 full-texts that were screened, 13 articles were included [3,15,16,17,18,19,20,21,22,23,24,25,26]. An additional four articles [27,28,29,30] were identified by reference list screening of the included articles, resulting in a total of 17 included studies (Figure 1).

### Characteristics of the included studies

Included studies, published between 2004 and 2019, were conducted in the United States (n = 8), Australia (n = 4), Denmark (n = 2), France (n = 1), Singapore (n = 1) and the Netherlands (n = 1) (Table 1). The total sample size per study ranged from 107 to 8,936 patients. The mean age ranged from 75.1 to 82.6 years in the intervention group and from 74.4 to 82.8 years in the control group respectively. A total of 15 different TCMs were studied, as three studies implemented the same TCM in different study populations [17,27,30]. Authors were not contacted for additional information, however for six TCMs additional information on methods and intervention was provided in previous publications [31,32,33,34,35,36].

### Description of the TCMs using SELFIE framework

#### Service delivery

All fifteen TCMs (Table 2) provided person-centred and tailored care. Fourteen models conducted a holistic tailored assessment, while one focused on providing a detailed medication reconciliation review [22]. Self-management was promoted in 12 out of 15 care models, mostly by providing education or exercise training for the patient. The TCM design by Coleman and colleagues encourages self-management and provides various tailored self-management methods to patients such as helping the patient to understand medications used, encouraging the patient or caregiver to make doctor appointments and rehearsing for upcoming appointments in order to make sure that needs are clearly articulated and teaching the patient to understand red flags regarding their health condition [17,27,30]. Informal caregivers were involved in nine studies by means of education (n = 3), involvement in care planning (n = 5) or focusing on caregiver burden during a home visit (n = 1). Checking for treatment interactions by reviewing medications for patients was conducted in all but one TCM. One study provided additional training to educate their coordinators on the interaction of heart failure and common comorbidities in older patients [21]. Continuity of care was provided in all TCMs, either by follow-up at home (n = 11), by telephone calls (n = 2) or outpatient clinic follow-up (n = 2). Eight TCMs offered intensive follow-up after discharge by combining two approaches: providing at least one home visit and telephone follow-up (n = 7) or conducting an outpatient clinic visit and telephone follow-up (n = 1). Follow-up after discharge was often provided timely within 24–96 hours [3,16,17,19,20,21,22,24,25,27,30].

#### Leadership and governance

Shared decision making to create an individualized care plan was part of seven TCMs. Mostly the patients and the families were involved in the process (n = 5), while two studies included the families only. Fourteen TCMs provided individual tailored care by assessing patient needs and planning appropriate follow-up care, whereas Simpson et al. [24] used a standardized set of ‘Hospital Elder Life Program’ (HELP) protocols that were delivered to the patient at the hospital and at home.

#### Workforce

A coordinator was named in 12 TCMs. While three TCMs mentioned several professional profiles that may assume coordination [24,26,28], other included studies specified a nurse (n = 8) or a pharmacist (n = 1) as coordinator. Coordinators responsibilities typically consisted of overseeing the discharge planning at the hospital, collaborating with physicians and other care providers, conducting follow-up visits/calls, addressing patient/caregivers concerns and organizing care services if needed. In addition to the coordinator, 11 models mentioned a core group of professionals who had clearly defined responsibilities and were mainly involved in providing transitional care. The core group consisted out of various professionals including nurses (n = 9), GPs (n = 2), geriatricians (n = 3), physiotherapists (n = 2), physician (n = 1), pharmacist (n = 1) therapist (n = 1) and clinician leader (n = 1). Nine TCMs specifically mentioned that a multidisciplinary team was involved in delivering transitional care to the patient.

While most studies specified the additional workforce needed in order to deliver the TCM components, the funding mechanisms of additional workforce was rarely reported.

#### Financing

The micro level of the SELFIE framework asks for the description of coverage and reimbursement structures of the TCMs. Only one included study reported on this topic. The coordinators of the PAC program had the flexibility to purchase services for patients with a dedicated budget in the immediate post-discharge period [29]. Providing financial structures that give enough flexibility to the provider are often stressed as important for integrated care programmes [19,29].

#### Technologies and medical products

Electronic medical records (EMRs) were used in five TCMs to facilitate communication between health care providers. In two studies the EMRs were additionally used to inform the study coordinator about new patient admissions [22,29]. Several assistive technologies were used to facilitate the TCMs. Two studies included assistive technologies that promoted the patients’ ability to participate in the follow-up care at home by providing a pedometer to report steps and distance in a diary [3] and audiotaping the patients’ teaching sessions in order for patients and caregivers to review the tapes with provided recorders throughout the intervention [21]. One study used an algorithm to create a list of evidence-based recommendations for each patient by using a web-based database that covered all documented patient information [23], while another study promoted professional communication by including the nurse practitioner contact information in the hospital-wide text paging system [29].

#### Information and Research

Thirteen TCMs shared individual healthcare data to effectively plan continuity of care of older patients. Out of those, ten TCMs shared medical information with the patient’s GP. Individual risk prediction was fulfilled by all as each TCM used the collected data to organize pro-active care and early treatment of identified risk factors.

### Reported outcomes

Twenty-seven different outcomes were measured within the included studies (Table 3). The outcomes hospital readmission (n = 17), mortality (n = 10), ED visits (n = 9), length of stay (n = 6) and total intervention costs (n = 6) were most frequently reported. Over the past 20 years (2004–2019) in all three continents (US, EU & APAC) these core outcomes have been consistently used to study the impact of TCMs. A difference is visible in cost outcomes including health care costs and total intervention costs as both are mainly reported in studies conducted in the US while no European studies reported cost outcomes.

Service outcomes are more frequently reported compared to patient and provider outcomes, as out of the ten most frequent reported outcomes, eight are service outcomes and two are patient outcomes namely mortality (n = 10) and health related quality of life (n = 5). Provider outcomes, such as caregiver burden (n = 1) and provider feedback (n = 1), are rarely reported.

Finally, 13 out of 17 articles reported on process outcomes and mostly measured adherence to the total intervention protocol or single intervention components.

### Impact on hospital readmission and ED visit outcomes

The implementation of five TCMs resulted in a significant reduction in hospital readmissions or ED visits [3,17,21,22,26,27,30] (Table 4). Four TCMs significantly reduced hospital readmissions at different post-discharge measurement moments namely at 15 days [26], at one month [17,26,27], at three months [17,27,30], at five to six months [3,17,26], and at one year after discharge [21].

Three TCMs showed significantly lower ED visit rates at one month after discharge [22,26], at three months after discharge [17] and at six months after discharge [26].

### SELFIE components used in effective TCMs

The SELFIE concepts service delivery, leadership & governance, workforce and information & research are described in the five TCMs that had a significant impact on hospital readmissions and ED visits (Table 2). Several components that are part of the SELFIE concepts such as Informal caregiver involvement, shared decision making intensive post-discharge care continuity and involvement of a project coordinator appear frequently in the five TCMs.

Informal caregivers were involved in all five models. Shared decision making processes involving informal caregivers was promoted in four out of five TCMs. Additionally four out of five TCMs provided intensive continuity of care by conducting at least two post-discharge follow-up methods such as a home visit and an additional telephone call. A coordinator with a nursing background was named in four effective TCMs, while one TCM defined a pharmacist as coordinator. Further, a small core group of professionals was defined in four TCMs. The use of technology is limited as only one TCM worked with an EMR system and assistive technologies were implemented in two TCMs.

Among the ten TCMs that did not show an impact on hospital readmission and ED visits, the SELFIE components informal caregiver involvement, shared decision making and intensive continuity of follow-up care were sparingly used. Informal caregiver involvement was used in four out of ten TCMs and shared decision making and intensive continuity of follow-up care were provided in three out of ten TCMs. Noticeably, the involvement of a multidisciplinary care team was described more frequently in eight out of ten TCMs.

## Discussion

In this systematic review including 17 studies and covering 15 TCMs, we summarize the integrated care components used, provide an overview on reported outcomes and describe the impact of TCM components on readmission and ED visit outcomes.

Our first objective to summarize intervention components indicates that all reviewed TCMs cover the SELFIE concepts of service delivery, leadership & governance, workforce and information & research. The concept of service delivery is best described in the included studies as all TCMs offered a person-centred, tailored, pro-active and continuous transitional care service by including pre-discharge and post-discharge components. Moreover, a broad range of health care professionals were involved in conducting the TCMs as the models either defined a coordinator (n = 12) and/or a core group (n = 9) and/or specified that the intervention was conducted in a multidisciplinary team (n = 9). Noticeable variations across the TCMs were identified in the involvement of the caregivers, the shared decision making process, and the intensity of follow-up care. Financing structures were rarely specified. Moreover, the use of technologies and medical products was scarce.

We observe a meaningful difference between TCMs in terms of the intensity of post-discharge follow-up provided. Eight TCMs conducted an intensive follow-up of combining a home visit or an outpatient clinic visit with a telephone follow-up, of which four were able to show a signification reduction in hospital readmission or ED visits for frail patients [3,17,21,26,27,30]. This confirms the conclusion of Morkisch et al. [10] that high intensity post-discharge follow-up is important to create impact. The researchers rated the intervention intensity of included trials by using a qualitative assessment and found that high intensive interventions were associated with reduced hospital readmission rates. The combination of follow-up types was one of the seven parameters included in the qualitative assessment [37].

Self-management was promoted in 12 TCMs by providing education or exercise trainings for patients, but a detailed description of what the self-management component entails was rarely provided. Leijten et al. [14] pointed out that self-management should be tailored to an individual starting point as multi-morbid patients may find self-management demanding. Additionally patient knowledge and motivation are important for successful self-management [19]. Hence, it is crucial for care providers to have a holistic understanding of the frail patient in order to individually assess self-management abilities and to provide education, support and encouragement as needed.

Another crucial aspect of successful TCMs is to define a coordinator [3,10,12]. Reviewed studies mostly named a professional with a nursing background as coordinator [3,15,16,17,19,20,21,27,29,30] and two TCMs appointed nurse coordinators with a master training [17,21,27,30]. Moreover, six TCMs mentioned that they provided additional education for coordinators and three TCMs stated that their coordinators were experienced in geriatric care. Leijten et al. [14] also highlighted that clear understanding of roles and responsibilities is critical for all people involved, which is supported by defining a coordinator who facilitated communication for providers between both settings and provided a safety net for patients and informal caregivers to address concerns. Hence, a small sized care team which is tailored and educated for the needs of the target population is best suited to provide transitional care for frail older patients.

GPs were involved in ten TCMs by means of sharing discharge plans, planning follow-up appointments for patients, sharing recommendations, giving advice or supporting the implementation of recommendations. Involving GPs in the transitional care process and sharing individual level data like discharge plans is recognized as important for successful continuity of care. However, several reviews have identified the high workload and lack of integrated computer systems as a challenge for fully involving GPs [12,38].

Technologies and medical products were scarcely used within the reviewed TCMs, as five TCMs included EMRs in their models and four studies included assistive technologies. While EMR systems provide a digital version of patient charts and enhance up-to-date information sharing within the same setting, electronic health records (EHRs) aim to facilitate communication between health care settings and to provide a holistic view of the patients’ health [39]. Most reviewed TCMs worked with the EMRs within the hospital setting. One TCM described their efforts to improve documentation between hospital staff and primary care providers by having a nurse practitioner as liaison person in the hospital that documented patient details during hospitalization in both EMR systems as each setting was using a different system [29]. However, none of the reviewed TCMs described the use of EHRs between hospital and home care setting, which clearly confirms the need to create a stronger digital link between settings and providers.

Nine TCMs involved informal caregivers in their intervention design and five TCMs specifically involved informal caregivers in the shared decision making process. Informal caregivers were included in discussing the patients’ health situations with practitioners and patients. Involving informal caregivers in developing care plans is common for older frail patients and becomes central when patients are less able to participate in the discussions [40]. Leijten et al. [14] points out that informal caregiver involvement is desirable whenever possible, but also states that caregiver burden should be considered. While most TCMs involved informal caregivers, only one study reported on caregiver burden [28]. This confirms that the gap identified in the review of Allen et al. [12] is still present and that a patient and family centred focus in future studies is urgently needed.

As a second objective the review provides an overview of outcomes reported within the included studies. A total of 27 outcomes were reported, from which 19 are categorized as service outcomes, six as patient outcomes and two as provider outcomes. While service outcomes were frequently reported, less attention was paid to patient and provider outcomes. This may be due to the fact that patient outcomes such as health related quality of life and patient satisfaction and provider outcomes such as caregiver burden are qualitative in nature and hence can be hard to measure as they require tailored tools to quantitatively scale them [41]. However, where data collection is feasible, paying equal attention to all three categories, namely, service, patient and provider outcomes can lead to a comprehensive assessment and provide a holistic view of the transitional care program.

Finally, We observed similarities in terms of integrated care components in the five TCMs that significantly impacted readmission or ED visit rates. Our findings suggest that intensive follow-up care, informal caregiver involvement, shared decision making and a small care team with a defined care coordinator increase the success of transitional care for frail older patients. Noticeable, four effective TCMs did not work with EMRs, suggesting EMRs not to be a necessary perquisite. A similar finding is stated by Leijten et al. [14] and Kansagara et al. [9], however the need for more technology development which is user-friendly and care process supportive is stressed. Moreover, the results clearly state a need to involve patients and caregivers in the TCM design through shared decision making as it allows to meet their needs and develop a care plan most beneficial to them [12].

Some limitations need to be addressed. Potential relevant studies might have been missed as no clinical trial databases were searched and no citation search was performed. However, a comprehensive search string was developed and references of all included papers were systematically checked. In addition, two TCMs that are included in this systematic review provide a very limited description of their intervention. This has complicated the data extraction, interpretation and mapping of components. We also observed that hospital readmission was reported as both planned, unplanned or a combination of both. The heterogeneity identified in the study demographics, methods and outcomes reported was also the reason why we did not perform a meta-analysis. Next, this review focused on mapping outcomes of studies at least reporting on hospital readmission and ED visit outcomes and did exclude qualitative studies providing valuable insights such as patient and provider experiences. The list of outcomes might therefore be incomplete. Finally, future reviews focusing solely on the effectiveness of TCMs should conduct a risk of bias assessment to consider the reported effects.

To conclude, findings of this systematic review suggest that a TCM which includes multi-components namely pre-discharge and intensive post discharge follow-up components can reduce hospital readmission and ED visits. Components like shared decision making, involvement of informal caregiver and a small tailored care team with a defined coordinator can increase the success of TCMs. However, the detected heterogeneity in TCMs as well as poor reporting of the meso and macro level and missing details of reporting in the micro level of the SELFIE framework allows no generalized conclusion. Future research should focus on strong methodological designs providing detailed reporting on TCM components and stronger and more process evaluations in order to understand the impact of individual components within these multilevel complex interventions and therefore to define clear recommendations for practice. Moreover, gaps in patient and provider outcome measures have been identified and need a stronger focus in future research.

## Additional Files

The additional files for this article can be found as follows:

10.5334/ijic.6447.s1 Supplementary File 1. Electronic search terms per database.

10.5334/ijic.6447.s2 Supplementary File 2. PRISMA 2020 Checklist.
