# Supplementary material for: Integrated Care Components in Transitional Care Models from Hospital to Home for Frail Older Adults: A Systematic Review
Source: Int J Integr Care. 2022 Jun 29;22(2):28. doi: 10.5334/ijic.6447 (PMC9248982; doi:10.5334/ijic.6447)
Supplement: Supplementary File 1. — Electronic search terms per database. [file ijic-22-2-6447-s1.pdf]

Supplementary file 1. Electronic search terms per database

Date when search was performed: July 30, 2020

NUMBER	PUBMED	EMBASE	CINAHL
**1**	frail elderly [MeSH]	‘Frail elderly’/exp	(MH "Frail Elderly")
**2**	geriatrics [MeSH]	‘geriatrics’/exp	(MH "Geriatrics")
**3**	multimorbidity [MeSH]	‘multiple chronic conditions’/exp	(MH "Chronic Disease+")
**4**	elder [Title/Abstract]	elder:ti,ab,kw	TI elder
**5**	frail senior [Title/Abstract]	Frail:ti,ab,kw	AB elder
**6**	geriatr* [Title/Abstract]	senior:ti,ab,kw	TI frail senior
**7**	multimorbid* [Title/Abstract]	geriatr*:ti,ab,kw	AB frail senior
**8**	Transitional care [MeSH]	multimorbid*:ti,ab,kw	TI geriatr*
**9**	continuity of patient care [MeSH]	‘transitional care’/exp	AB geriatr*
**10**	delivery of health care, integrated [MeSH]	‘patient care’/exp	TI multimorbid*
**11**	care continuity [Title/Abstract]	‘integrated health care system’/exp	AB multimorbid*
**12**	continuity [Title/Abstract]	care AND continuity:ti,ab,kw	(MH "Transitional Care")
**13**	transition [Title/Abstract]	continuity:ab,ti,kw	(MH "Continuity of Patient Care+")
**14**	transitional care [Title/Abstract]	transition:ti,ab,kw	(MH "Health Care Delivery, Integrated")
**15**	integrated care [Title/Abstract]	transitional AND care:ti,ab,kw	TI care continuity
**16**	hospitalization [MeSH]	integrated AND care:ti,ab,kw	AB care continuity
**17**	hospitals [Title/Abstract]	'hospitalization'/exp	TI continuity
**18**	hospitalization [Title/Abstract]	Hospitals:ti,ab,kw	AB continuity
**19**	patient discharge [Title/Abstract]	hospitalization:ti,ab,kw	TI transition

**20**	OR/1-7	Patient AND discharge:ti,ab,kw	AB transition
**21**	OR/8-15	OR/1-8	TI transitional care
**22**	OR/16-19	OR/9-16	AB transitional care
**23**	20 AND 21 AND 22	OR/17-20	TI integrated care
**24**	AND (English[Language]) OR (Dutch[Language]) OR (German[Language])	21 AND 22 AND 23	AB integrated care
**25**	AND ("2000/01/01"[Date - Publication] : "2020/06/01"[Date - Publication])	AND german:la OR dutch:la OR english:la	(MH "Hospitalization+")
**26**		AND [1-1-2000]/sd NOT [2-6-2020]/sd	TI hospitals
**27**			AB hospitals
**28**			TI hospitalization
**29**			AB hospitalization
**30**			TI patient discharge
**31**			AB patient discharge
**32**			OR/ 1-11
**33**			OR/12-24
**34**			OR/25-31
**35**			32 AND 33 AND 34
**36**			AND LA english OR LA dutch OR LA german
**37**			AND Publication Date limitation
